# Monoclonal antibody pairs against SARS-CoV-2 for rapid antigen test development

**DOI:** 10.1371/journal.pntd.0010311

**Published:** 2022-03-31

**Authors:** Nol Salcedo, Ankita Reddy, Adam R. Gomez, Irene Bosch, Bobby Brooke Herrera

**Affiliations:** 1 E25Bio, Inc., Cambridge, Massachusetts, United States of America; 2 Perelman School of Medicine, University of Pennsylvania, Philadelphia, Pennsylvania, United States of America; 3 Department of Medicine, Mount Sinai School of Medicine, New York, New York, United States of America; 4 Institute for Medical Engineering and Science, Massachusetts Institute of Technology, Cambridge, Massachusetts, United States of America; 5 Department of Immunology and Infectious Diseases, Harvard T.H. Chan School of Public Health, Boston, Massachusetts, United States of America; George Washington University School of Medicine and Health Sciences, UNITED STATES

## Abstract

**Background:**

The focus on laboratory-based diagnosis of coronavirus disease 2019 (COVID-19) warrants alternative public health tools such as rapid antigen tests. While there are a number of commercially available antigen tests to detect severe acute respiratory syndrome coronavirus 2 (SARS-CoV-2), all cross-react with the genetically similar SARS-CoV-1 or require an instrument for results interpretation.

**Methodology/Principal findings:**

We developed and validated rapid antigen tests that use pairs of murine-derived monoclonal antibodies (mAbs), along with gold nanoparticles, to detect SARS-CoV-2 with or without cross-reaction to SARS-CoV-1 and other coronaviruses. In this development, we demonstrate a robust antibody screening methodology for the selection of mAb pairs that can recognize SARS-CoV-2 spike (S) and nucleocapsid (N) proteins. Linear epitope mapping of the mAbs helped elucidate SARS-CoV-2 S and N interactions in lateral flow chromatography. A candidate rapid antigen test for SARS-CoV-2 N was validated using nasal swab specimens that were confirmed positive or negative by quantitative reverse-transcription polymerase chain reaction (RT-PCR). Test results were image-captured using a mobile phone and normalized signal pixel intensities were calculated; signal intensities were inversely correlated to RT-PCR cycle threshold (Ct) value.

**Conclusion/Significance:**

Overall, our results suggest that the rapid antigen test is optimized to detect SARS-CoV-2 N during the acute phase of COVID-19. The rapid antigen tests developed in this study are alternative tools for wide scale public health surveillance of COVID-19.

## Author summary

The delays in diagnostic testing and lack of proper surveillance during the COVID-19 pandemic have contributed, in part, to the unprecedented death toll and impediment to wellbeing. As asymptomatic individuals have contributed to a large portion of disease spread, improved public health tools for widespread screening are necessary to maintain low transmission levels. We developed new rapid antigen tests that can be administered in less than 15 minutes without instrumentation, offering potential for frequent asymptomatic screening at-home and for point-of-care use in high-traffic areas such as schools, hospitals, and airports. The SARS-CoV-2 monoclonal antibody screening methodology resulted in pairs with or without cross-reaction to SARS-CoV-1, offering new opportunities for public health tools such as wide scale surveillance. This paper provides important insights for nanoparticle-based immunochromatographic assays.

## Introduction

The coronavirus disease 2019 (COVID-19) pandemic has resulted in an unprecedented public health crisis. As of October 2021, there have been approximately 245 million confirmed cases globally dealing devastating effects to economies and livelihood [1–3]. Individuals experiencing asymptomatic infection are a main source for the spread of COVID-19, with epidemiological and modeling studies estimating the number of cases to be more than 6 times greater than what is accounted for by surveillance systems [4–9]. As governments relax COVID-19 restrictions and societies begin to re-open, the ability to detect the disease as soon as it arises in communities is critical to maintaining low transmission levels.

The etiologic agent of COVID-19 is severe acute respiratory syndrome coronavirus 2 (SARS-CoV-2). The virus contains an enveloped single-stranded, positive-sense 30 kb RNA genome, which encodes structural proteins (membrane, M; envelope, E; spike, S; and nucleocapsid, N) and nonstructural proteins [10]. The viral membrane, consisting of M, E, and S, surrounds a helical N, in which the viral genome is encapsulated by the N protein [11,12]. The S and N proteins of SARS-CoV-2 are produced and secreted at high concentrations in the oropharynx and nasopharynx during infection, making them ideal diagnostic targets [13]. With the ongoing worldwide transmission of SARS-CoV-2 and emergent variants, there is a pressing need for large scale distribution of easily deployable, low-cost rapid tests.

A multitude of diagnostic approaches have been developed throughout the course of the pandemic, such as chest imaging, virus isolation, and molecular, serological, and antigen testing [14,15]. Molecular tests such as reverse-transcription polymerase chain reaction (RT-PCR) is the clinical gold-standard method for laboratory diagnosis and can detect viral nucleic acids during the acute phase of infection, unlike serological methods based on human IgG and IgM [16,17]. While RT-PCR tests often perform with high sensitivity and specificity, they require expensive instrumentation and reagents, experienced personnel, and have lengthy processing times [18]. As RT-PCR has failed to meet the global demands for rapid and early detection, recent studies have demonstrated that the frequency of testing, enabled by accessibility and turnaround time, should be prioritized for effective control of SARS-CoV-2 transmission [19–22].

Rapid antigen tests address shortcomings of the aforementioned diagnostics and provide large-scale screening implications. Rather than detecting nucleic acids or human antibodies, antigen testing detects the viral proteins, allowing for detection of an active infection with relative ease of sample collection and assay. These rapid tests can be mass-produced at low cost and administered by the average person without a laboratory or instrumentation. These tests also take 15 minutes or less to determine the result, enabling real-time diagnosis and surveillance.

In this paper, we demonstrate a robust antibody screening method to isolate murine monoclonal antibodies (mAbs) against SARS-CoV-2 S and N proteins. We then describe the development and validation of rapid antigen tests that use combinations of the mAbs, along with gold nanoparticles, to detect SARS-CoV-2 with or without cross-reactivity to the genetically related severe acute respiratory syndrome coronavirus 1 (SARS-CoV-1). Our results show robust detection of SARS-CoV-2 S and N proteins using recombinant antigens, virus infected cells, and nasal swab specimens from patients experiencing acute COVID-19. We offer these reagents and technologies as public health tools, especially for frequent use and surveillance of COVID-19.

## Methods

### Ethics statement

SARS-CoV-2 S1 and N mAbs were produced in mice under contract (Covance, Inc., Denver, PA), following an approved animal care protocol (Institutional Animal Care and Use Committee number 0016–20). Additionally, the primary studies under which the clinical samples were collected received ethical clearance from the PATH (www.path.org) Institutional Review Board (IRB) (approval number 00004244). Formal written consent was obtained from all participants, all excess samples and corresponding data were banked and de-identified prior to analyses. This study received an exemption determination from the PATH IRB.

### Monoclonal antibody production strategy

BALB/c mice were immunized with purified SARS-CoV-2 S1 (Catalog number 40591-V08H, SinoBiological, China) and N (Catalog number 40588-V08B, SinoBiological, China) proteins that were expressed in eukaryotic cells to facilitate proper protein folding and post-translational modifications. From each of the immunized mice, seroconverted animals with high titers of antibodies recognizing SARS-CoV-2 S1 or N proteins were used for cell fusion to generate hybridomas. Supernatants from cloned hybridomas were tested by ELISA to generate “fold over background” values. Supernatants were also used to evaluate relative binding to MERS (Catalog number 40069-V08B1, SinoBiological, China) and the human coronaviruses NL63 (Catalog number 40600-V08H, SinoBiological, China), 229E (Catalog number 40601-V08H, SinoBiological, China), HKU1 (Catalog number 40021-V08H, SinoBiological, China), and OC43 (Catalog number 40607-V08H1, SinoBiological, China). Positive reactions were identified by counting the clones that were equal to or greater than the threshold value (value of the blank plus 5 times the standard deviation of the blank signal intensity).

The candidate SARS-CoV-2 S1 and N hybridomas were expanded by growing in low IgG serum (Catalog number 16250078, ThermoFisher Scientific, Waltham, MA) containing hybridoma cloning supplement (Catalog number 11363735001, MilliporeSigma, Burlington, MA). The expressed mAbs were isotyped using IsoStrip Mouse Monoclonal Antibody Isotyping Kit (Catalog number 11493027001, MilliporeSigma, Burlington, MA) according to the manufacturer’s instructions and purified by affinity chromatography on a protein L matrix (Catalog number 17547801, Cytiva, Marlborough, MA). Purified mAbs were tested pairwise in immunochromatography paperfluidic tests (E25Bio, Inc., Cambridge, MA) to identify pairs that exhibited high binding to SARS-CoV-2 S1 or N proteins and low to no non-specific binding.

### Flow cytometry analysis of the hybridoma supernatants

To ensure that the mAbs expressed by hybridomas recognized native SARS-CoV-2 S1 and N proteins, hybridoma supernatants were used as a source of anti-S1 and anti-N antibodies for immunostaining Vero cells that had been infected with SARS-CoV-2, human coronaviruses 229E and OC43, or non-infected cells as a control. Vero cells (90% confluency) were infected with these viruses at multiplicity of infection (MOI) of 1 and incubated for 24 hours. The cells (50,000 cells per well of a 96-well plate) were prepared for immunostaining by using the BD Cytofix/Cytoperm Fixation/Permeabilization Solution Kit (Catalog number 554714, ThermoFisher Scientific, Waltham, MA) according to the manufacturer’s instructions. Briefly, washed and fixed cells were incubated with 100 μl of primary mouse hybridoma media for 1 hour, washed and incubated for 1 hour with phycoerythrin (PE)-labeled anti-mouse secondary antibody (Catalog number P-852, ThermoFisher Scientific, Waltham, MA). Immuno-reacted cells were analyzed using an Attune NxT Flow Cytometer (ThermoFisher Scientific, Waltham, MA). Live cells were gated as previously described, and fluorescence was quantified using a positive control anti-SARS-CoV-2 S1 (Catalog number 40150-D002, SinoBiological, China) and N (Catalog number 40143-R001, SinoBiological, China) antibodies.

### Antibody conjugation to nanoparticles

The SARS-CoV-2 S1 and N mAbs were conjugated to gold nanoparticles (Catalog number ab154873, Abcam, Cambridge, UK) according to the manufacturer’s instructions. Briefly, the mAb was first diluted to 0.2 mg/ml in the supplied dilution buffer. Next, 12 ul of diluted antibody was mixed with 42 μl reaction buffer. 45 μl of the mix was then used to suspend the lyophilized gold nanoparticles. The antibody-nanoparticle mix was incubated for 15 minutes at room temperature, followed by the addition of 5 μl of proprietary quencher solution to stop the coupling reaction. After adding the quencher solution, 100 ul of 1% Tween-20 (Catalog number P9416, MilliporeSigma, Burlington, MA) in PBS (Catalog number P4417, MilliporeSigma, Burlington, MA) and 50 μl of 50% sucrose (Catalog number S0389, MilliporeSigma, Burlington, MA) in water were added to the gold conjugates prior to use in immunochromatography.

### Antibody application to nitrocellulose membranes

Nitrocellulose membrane (Catalog number 1UN15LR100025NT, Sartorius, France) were cut into strips using a laser cutter (Universal Laser Systems; model VLS2.30; 30 watt) at 30% power and 90% speed. Strips were attached to a wick (Catalog number WHA10427818, MilliporeSigma, Burlington, MA) with adhesive paper (Catalog number, DCN Diagnostics, Carlsbad, CA). For the control area, 0.33 ul of anti-mouse Fc antibody (Catalog number M4280, MilliporeSigma, Burlington, MA) at 2 mg/ml was spotted on the control line. The anti-CoV-2 capture line on the nitrocellulose was generated by pipetting 0.33 μl of SARS-CoV-2 S1 or N mAbs at 4 mg/ml at the test area. Strips were air-dried and stored in a desiccator at room temperature before use.

### Immunochromatography

Each immunochromatography strip was run in a separate microcentrifuge tube, and groups of tubes/strips were run together. The rapid test solution contained 1) 50 μl of sample (e.g., recombinant protein, contrived nasal swab specimens, nasal swab specimens from acute COVID-19 patients, 2) 10 μl of fetal calf serum, 3) 5 ul of proprietary quencher solution (Abcam, Cambridge, UK), and 4) 15 ul of conjugated gold nanoparticle mix. The run time varied between 15–20 minutes. After the strips dried, results were image captured for signal pixel intensity analysis.

### Epitope mapping

Linear epitope mapping was used for further antibody characterization and also to inform the interpretation of the paired antibody ELISA assays. Linear epitopes were analyzed by ELISA method. Libraries of tiled SARS-CoV-2 S1 (Catalog number NR-52418, BEI Resources, Manassas, VA) and N (Catalog number NR-52419, BEI Resources, Manassas, VA) peptides were resuspended according to manufacturer’s instructions. Peptides were diluted to a working concentration of 100 ug/ml in water. Each well (10 μg of each peptide) was diluted in 50 μL of ddH_2_O and incubated at 37°C until complete evaporation of the liquid. The plates were blocked with 200 μL/well of PBS and 5% (blocking buffer) (Catalog number A9418, MilliporeSigma, Burlington, MA) for 2 hours at 37°C. The plates were rinsed 3 times with PBS and 0.05% Tween-20 and incubated with 100 μL/well of primary antibody in a blocking buffer (at 10 μg/mL) for 1 hour at 37°C. The plates were washed 3 times with PBS and 0.05% Tween-20. Anti-mouse HRP-conjugated IgG antibody (Catalog number ab6789, Abcam, Cambridge, UK) was used as a secondary (diluted 1:5000) 100 μL/well for 30 minutes at 37°C. After another 3 washes, 100 μL/well of TMB Chromagen Solution (Catalog number 002023, ThermoFisher Scientific, Waltham, MA) was applied to each well for 5–10 minutes. The reaction was terminated using 50 μL of 0.5 M H_2_SO_4_ and absorbance was measured at 450 nm with a GloMax Explorer Multimode Microplate Reader (Promega, Madison, WI).

### Clinical samples

The study included nasal swab specimens provided by the non-profit PATH. The panel nasal swab specimen dilutions were prepared from human nasal swab eluate discards in PBS. Swab eluates positive for SARS-CoV-2 by RT-PCR from multiple individuals were combined and diluted into nasal swab eluate pools from individuals testing negative for SARS-CoV-2 by RT-PCR. Dilutions were aliquoted and frozen at -80°C. Additionally, nasal swab specimens collected during the acute phase (between 1–7 days post-onset of fever) were collected, aliquoted and frozen at -80°C.

Aliquots were thawed and 200 μl was used for extraction with the QIAamp Viral RNA Mini Kit (Catalog number 52904, Qiagen, Germany). Nucleic acids were eluted in 50 μl and 10 μl were used for qRT-PCR using the CDC’s 2019-nCoV Real-Time RT-PCR N1 assay on the QuantStudio 5 Real-Time PCR System (Applied Biosystems, Foster City, CA). Viral loads of SARS-CoV-2 were determined by measuring the concentration of the RNA and N protein using previously qualified standards to determine genome RNA levels and N protein concentrations.

### Limits of detection

Limits of detection for each of the mAb pairs were measured using SARS-CoV-2 S1 and N proteins. Antigen solutions were diluted serially and chromatographed on the rapid antigen tests. The signal intensities were quantified in ImageJ (NIH), normalized by the intensity at the highest concentration, where gray_n_ = [1]/[gray_max_-gray_0_]. Gray_0_ represents the pixel signal intensity of the blank area, gray_max_ represents the pixel signal intensity of the highest concentration point (at saturation), and gray_n_ represents the pixel signal intensity at each concentration. After normalization, gray values were plotted and fitted in a Langmuir equation gray_n_ = [antigen]/[K_d_^effective^ + antigen], where[antigen] is the concentration of antigen present in the sample, and K_d_^effective^ is the effective binding constant in a Langmuir-like system. The limit of detection (LoD) was calculated from the curve fit as the concentration found at the intersection with a line representing the value of the blank plus 5 times the standard deviation of the blank signal intensity. Analysis was performed in duplicate for each curve.

### Western blotting

Briefly, SARS-CoV-2 S1 (Catalog number 40591-V08H, SinoBiological, China), S2 (Catalog number 40590-V02H, SinoBiological, China), and RBD (Catalog number 40592-V08B, SinoBiological, China) were added to reducing buffer and subjected to 12% PAGE and Western blot analysis using the 1:1000 of the mAbs (stock concentration 1 mg/ml) as primary antibody and anti-mouse IgG HRP (Catalog number ab6789, Abcam, Cambridge, UK) as secondary antibody. Visualization was performed using SuperSignal West Pico PLUS chemiluminescent substrate (Catalog number 34579, ThermoFisher Scientific, Waltham, MA) per the manufacturer’s instructions with the iBright FL1500 Imaging System (ThermoFisher Scientific, Waltham, MA).

### Image analysis

Rapid antigen tests results were analyzed using image processing software to machine-read and quantify signal pixel intensity results. The image of the nitrocellulose membrane was captured with a mobile phone camera and analyzed using an ImageJ macro to quantify test results. ImageJ is used as a public domain image analysis software. The same mobile phone camera was used throughout the study. ImageJ quantified the signal at the test area and blank/background area and generated a normalized intensity, calculated by dividing the maximum intensity value at the test area by the average value of a blank area on each rapid antigen test.

### Statistical analysis

GraphPad Prism 9.0 software was used to report the performance of the rapid test through the PCR comparison and the Receiver Operating Characteristic (ROC) curves. A simple linear regression analysis was performed on the Signal Intensity vs. PCR Ct data. The ROC curve presents test performance as True Positive Rate (% sensitivity) versus False Positive Rate (100%—% specificity). Optimal cutoff values, which maximize sensitivity and specificity, were calculated from the ROC curve also using GraphPad Prism 9.0. The sensitivity is defined as the fraction of total confirmed positive samples that are true positives according to the test. The specificity is defined as the fraction of total confirmed negative samples that are true negatives according to the test.

## Results

### Antibody selection for SARS-CoV-2 rapid antigen tests

mAbs that specifically recognize SARS-CoV-2 were unavailable during the early months of the COVID-19 pandemic, and a majority of the antibodies that were commercially available had been generated against SARS-CoV-1. Given the amino acid sequence homology of immune-dominant portions of Spike between SARS-CoV-1 and SARS-CoV-2 and overall homology of N protein, manufacturers were unable to develop and bring to market specific antigen-based tests that detect SARS-CoV-2 without cross-reaction to SARS-CoV-1. Of 25 SARS-CoV-2 antigen-based tests that have obtained an Emergency Use Authorization from the United States Food and Drug Administration (FDA), 24 cross-react with SARS-CoV-1; additionally, the one test that does not cross-react with SARS-CoV-1 requires an instrument for results interpretation and is therefore limited to laboratory diagnosis (S1 Table) [23]. Thus, we attempted to generate mAbs against SARS-CoV-2 spike subunit 1 (S1)—which contains the receptor binding domain (RBD)—and N.

**Fig 1:**
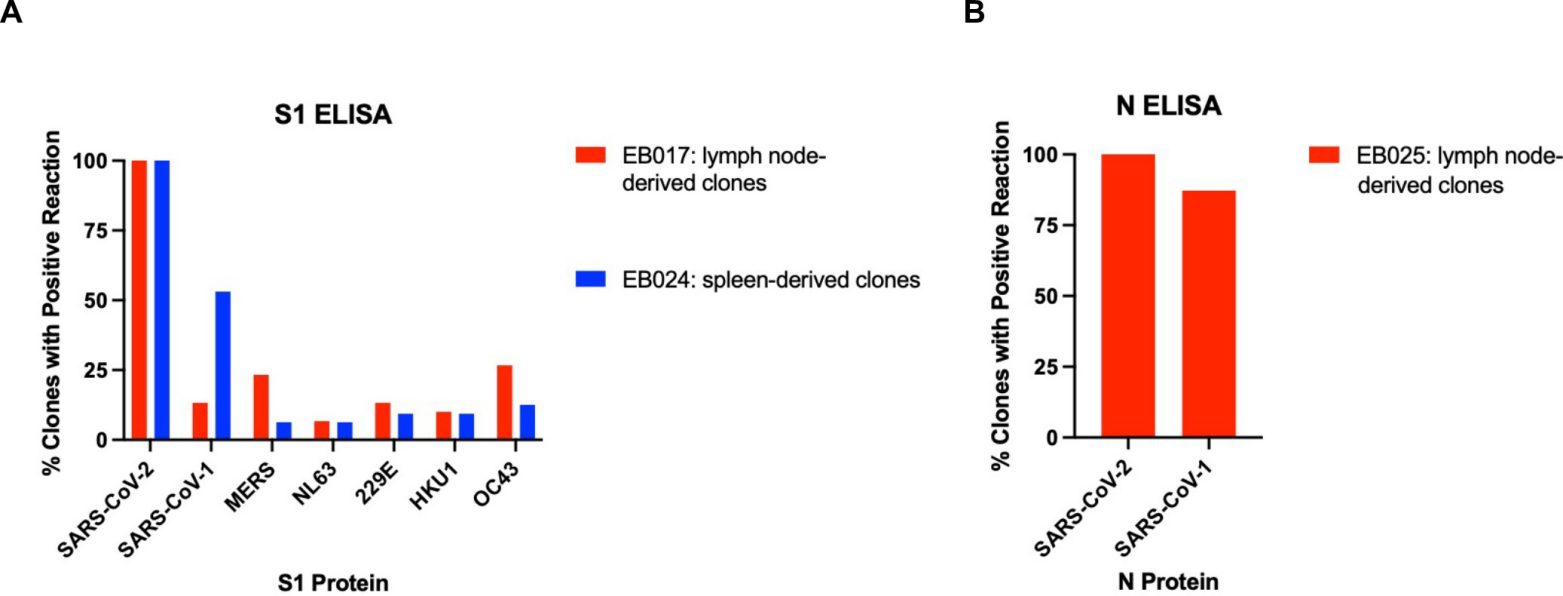
Binding of SARS-CoV-2 S1 and N mAbs. mAb clones were harvested from the hybridomas of SARS-CoV-2 spike subunit (S1) or nucleocapsid (N) immunized mice. (A) ELISA using hybridoma supernatants from the lymph nodes of mouse EB017 or the spleen of mouse EB024, both of which were infected with SARS-CoV-2 S1. Hybridoma supernatants were tested with S1 from SARS-CoV-2, SARS-CoV-1, MERS, NL63, 229E, HKU1, and OC43 to evaluate cross-reactivity. B) ELISA using hybridoma supernatants from the lymph nodes of mouse EB025 infected with SARS-CoV-2 N. Hybridoma supernatants were tested with S1 from SARS-CoV-2 and SARS-CoV-1 to evaluate cross-reactivity. Cross rection for both ELISAs is measured by % clones that demonstrated a positive reaction, or an OD_50_ greater than or equal to 5 times the standard deviation of the blank signal intensity.

Mice were injected separately with recombinant SARS-CoV-2 S1 or N proteins. B cells from the lymph node or spleen were fused with mouse myeloma cells to generate hybridomas. Relative binding to SARS-CoV-2 S1 or N proteins was analyzed by enzyme-linked immunosorbent assays (ELISA) using 60 hybridomas supernatants from mouse EB017 (S1-immunized, lymph node-derived), 46 hybridoma supernatants from mouse EB024 (S1-immunized, spleen-derived), and 47 hybridoma supernatants from mouse EB025 (N-immunized, lymph node-derived) (Fig 1). Relative binding to SARS-CoV-1 S1 or N proteins, Middle East respiratory syndrome coronavirus (MERS-CoV) S1 protein, and the human coronaviruses 229E, HKU1, NL62, or OC43 S1 proteins were analyzed in parallel by ELISA using the hybridoma supernatants to identify any potential cross-reactivity (Fig 1A and 1B). While 100% of the clones reacted to SARS-CoV-2 S1, 42/60 mAbs were specific only to SARS-CoV-2, and thus non cross-reactive to the six other S1 proteins (Fig 1A). The spleen-derived EB024 hybridoma supernatants screen demonstrated that all clones positively reacted with the SARS-CoV-2 S1 protein, with 15/46 mAbs demonstrating specificity (Fig 1A). Spleen-derived SARS-CoV-2 S1 hybridomas demonstrated a higher percent of mAbs that were cross-reactive with SARS-CoV-1 S1 (53.1%) proteins compared to lymph node-derived hybridomas (13.3%) (Fig 1A). Finally, lymph node-derived EB025 hybridoma supernatants demonstrated high reactivity to SARS-CoV-1 N, rendering 12.8% of clones being specific to SARS-CoV-2 N (Fig 1B).

Using the relative binding results from the ELISA, 78 spike-specific hybridoma supernatants were selected for further investigation. Hybridoma supernatants were used to stain permeabilized Vero cells that had been infected with either SARS-CoV-2, the human coronaviruses 229E or OC43, or non-infected cells as a control. Flow cytometric analysis demonstrated that the antibodies recognized SARS-CoV-2 S1 or N proteins expressed by virus-infected cells and provided a quantitative readout of potential cross-binding to Vero cells infected with the human coronaviruses 229E or OC43 (S1A–S1C Fig). A total of 43 hybridoma supernatants contained antibodies that recognized SARS-CoV-2 and/or SARS-CoV-1 S1 or N proteins without cross-reaction to other human coronaviruses; therefore, these hybridomas were expanded, and the mAbs were isotyped to allow for purification by affinity chromatography (Table 1).

**Table 1:** mAbs that detect SARS-CoV-2 S1 or N proteins by ELISA and flow cytometry.

	mAb	Hybridoma	Isotype
**SARS-CoV-2 S1**	1	**Lymph Nodes-derived (EB017)**	IgG1/Kappa
	5		IgG1/IgG3/Kappa
	46		IgG1/Kappa
	56		IgG1/IgG3/Kappa
	60		IgG1/Kappa
	74		IgG1/Kappa
	76		IgG1/Kappa
	80		IgG1/Kappa
	124		IgG1/Kappa
	129		IgG1/Kappa
	145		IgG1/Kappa
	168		IgG1/IgG2a/Kappa
	244		IgG1/IgG3/Kappa
	280		IgG1/Kappa
	296		IgG1/Kappa
	350		IgG1/Kappa
	359		IgG1/Kappa
	373		IgG1/Kappa
	374		IgG1/IgG3/Kappa
	376		IgG1/Kappa
	467		IgG1/Kappa
	474		IgG1/Kappa
	303	**Spleen-derived (EB024)**	IgG1/Kappa
	349		IgG1/Kappa
	502		IgG1/Kappa
	645		IgG1/Kappa
	854		IgG1/Kappa
	145		IgG1/Kappa
	469		IgG1/Kappa
	493		IgG1/Kappa
	571		IgG1/Kappa
**SARS-CoV-2 N**	1	**Lymph Node-derived (EB025)**	IgG1/Kappa
	79		IgG1/IgG3/Kappa
	134		IgG1/Kappa
	149		IgG1/Kappa
	189		IgG1/Kappa
	192		IgG1/Kappa
	267		IgG1/Kappa
	310		IgG1/Kappa
	329		IgG1/Kappa
	429		IgG1/Kappa
	448		IgG1/IgG2a/Kappa
	453		IgG1/Kappa

The purified mAbs were then tested in immunochromatography pairs, with one antibody conjugated to gold nanoparticles (gold conjugate) and one antibody absorbed to a nitrocellulose membrane. Testing throughput was maximized by placing nitrocellulose membranes in microcentrifuge tubes containing small-volume suspensions of the gold conjugate and antigen for 15 minutes, without the need for additional sample paper or conjugate pads that are characteristic of lateral flow chromatography. The 31 SARS-CoV-2 S1 mAbs and 12 SARS-CoV-2 N mAbs were tested in a matrix for interaction with SARS-CoV-2 S1 or N, as well as without any added antigen as a control. We tested 258 S1 mAb pairs (S2A Fig) and 126 N mAb pairs (S2B Fig). The pairwise chromatography analysis demonstrated a variety of signal intensities across the mAb combinations scored as either low binding (1, >201 normalized grey scale pixels), medium binding (2, 141–200 normalized grey scale pixels), or high binding (3, <140 normalized grey scale pixels), with a majority of the combinations presenting with no signal (white). Taken together, our screening method using ELISA, flow cytometry, and systematic pairwise immunochromatography identified mAbs that specifically recognize SARS-CoV-2 and/or SARS-CoV-1 S1 or N proteins without cross-reaction to other human coronaviruses.

### Limits of detection using recombinant proteins and characterized nasal swab specimens

**Fig 2:**
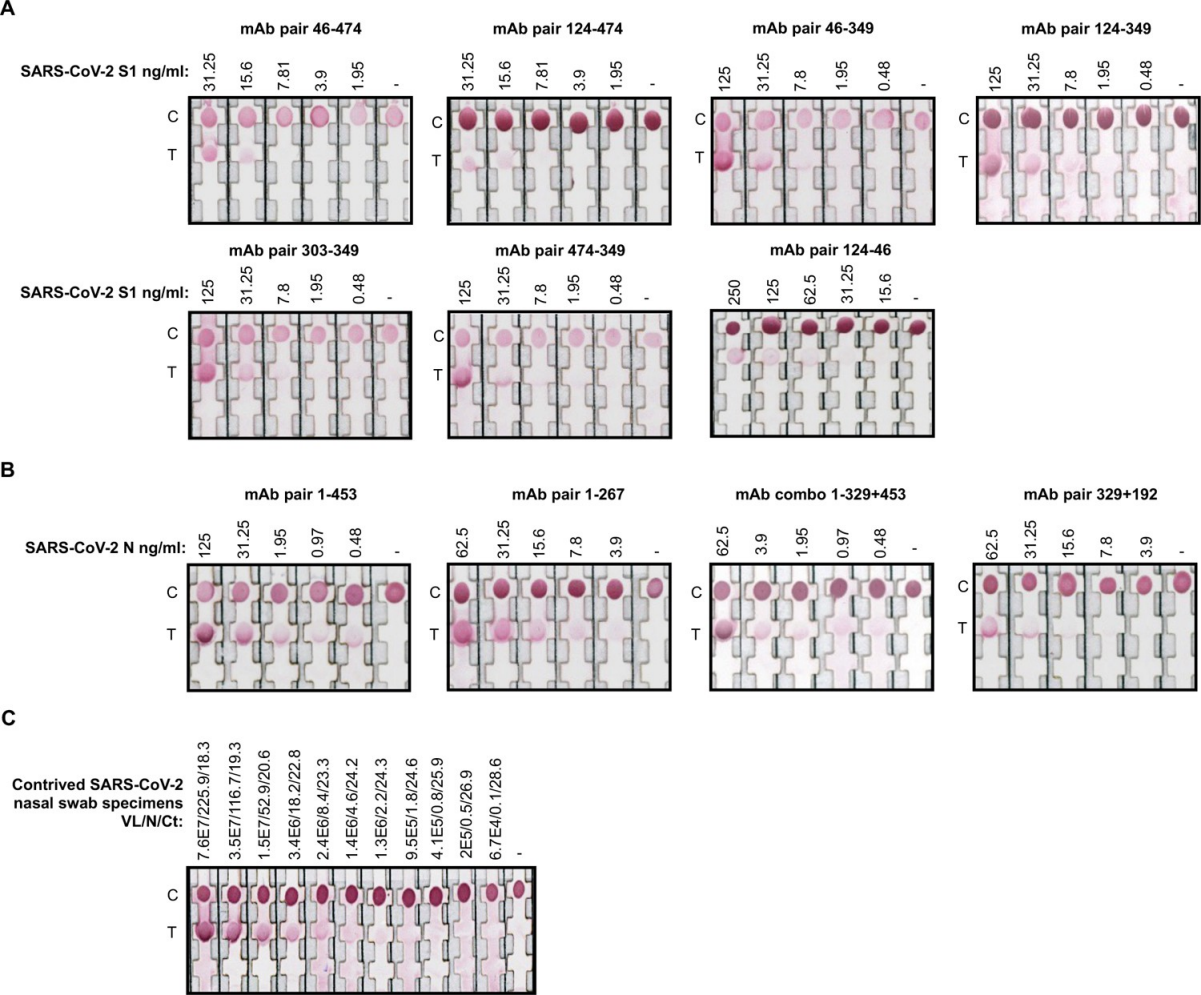
Limit of detections of mAb combinations. Serially diluted recombinant SARS-CoV-2 spike (S1) or nucleoprotein (N) were run on dipsticks with pairs of (A) SARS-CoV-2 S1 mAbs or (B) SARS-CoV-2 N mAbs. mAbs were either conjugated to the nanoparticle or applied onto the nitrocellulose membrane to create a sandwich immunoassay if antigen binding was present. (C) The SARS-CoV-2 N rapid test composed of mAbs 1 and 453 was selected based on its lowest limit of detection to evaluate performance using a panel of contrived SARS-CoV-2 nasal swab specimens. Nasal swab specimens with known viral loads (VL), nucleocapsid (N) protein concentrations, and cycle threshold (Ct) values were allowed to react with the rapid test for 15 minutes. C, control. T, test.

A total of 11 mAb pairs with high binding to SARS-CoV-2 S1 or N proteins were chosen to define limits of detection (LoD) and association and dissociation constants (K_a_ and K_d_). The LoD, K_a_ and K_d_, and standard deviations were determined by flowing serially diluted recombinant SARS-CoV-2 S1 or N proteins across the rapid antigen tests composed of selected S1 or N mAb pairs. After 15 minutes, the results from the rapid antigen tests were image captured and the normalized grey scale pixel intensities were quantified. The LoD for the SARS-CoV-2 S1 and N rapid antigen tests ranged between 4.89–19.38 ng/mL and 0.76–6.95 ng/mL, respectively (Fig 2A and 2B, Table 2).

**Table 2:** Limit of detection of mAb combinations.

	Gold Conjugate mAb	Nitrocellulose membrane mAb(s)	Association Constant (K_a_)	Dissociation Constant (K_d_)	Standard Deviation	Limit of Detection (ng/mL)
**SARS-CoV-2 S1**	46	474	0.06063	16.4939	0.054	7.07
	124	474	0.075125	13.31108	0.081	9.06
	46	349	0.039964	25.02281	0.034	5.12
	124	349	0.049574	20.17198	0.039	4.89
	303	349	0.043526	22.97455	0.038	5.39
	474	349	0.029976	33.36055	0.031	5.89
	124	46	0.009107	109.8084	0.030	19.38
**SARS-CoV-2 N**	1	453	0.09772	10.23341	0.016	0.76
	1	267	0.07641	13.08809	0.035	2.78
	1+329	453	0.13037	7.670434	0.018	0.89
	329	192	0.04418	22.63625	0.047	6.95

Our candidate SARS-CoV-2 N rapid antigen test composed of mAb 1 conjugated to gold nanoparticles and mAb 453 absorbed to the nitrocellulose membrane performed with the lowest LoD (0.76 ng/mL) across all other mAb pairs analyzed (Fig 2B). We further tested the LoD of this rapid antigen test using previously quantified nasal swab specimen dilutions in which the viral loads (SARS-CoV-2 counts/ml) and concentrations of SARS-CoV-2 N had been previously characterized [24]. Using these specimens, the LoD was 9.53E5 SARS-CoV-2 counts/ml and 1.8 ng/ml (standard deviation: 0.021) of SARS-CoV-2 N protein (Fig 2C). These results demonstrated the ability of the candidate rapid antigen test to robustly detect SARS-CoV-2 N using both recombinant antigen and contrived nasal swab specimen dilutions from patient samples.

### Linear epitope mapping

**Fig 3:**
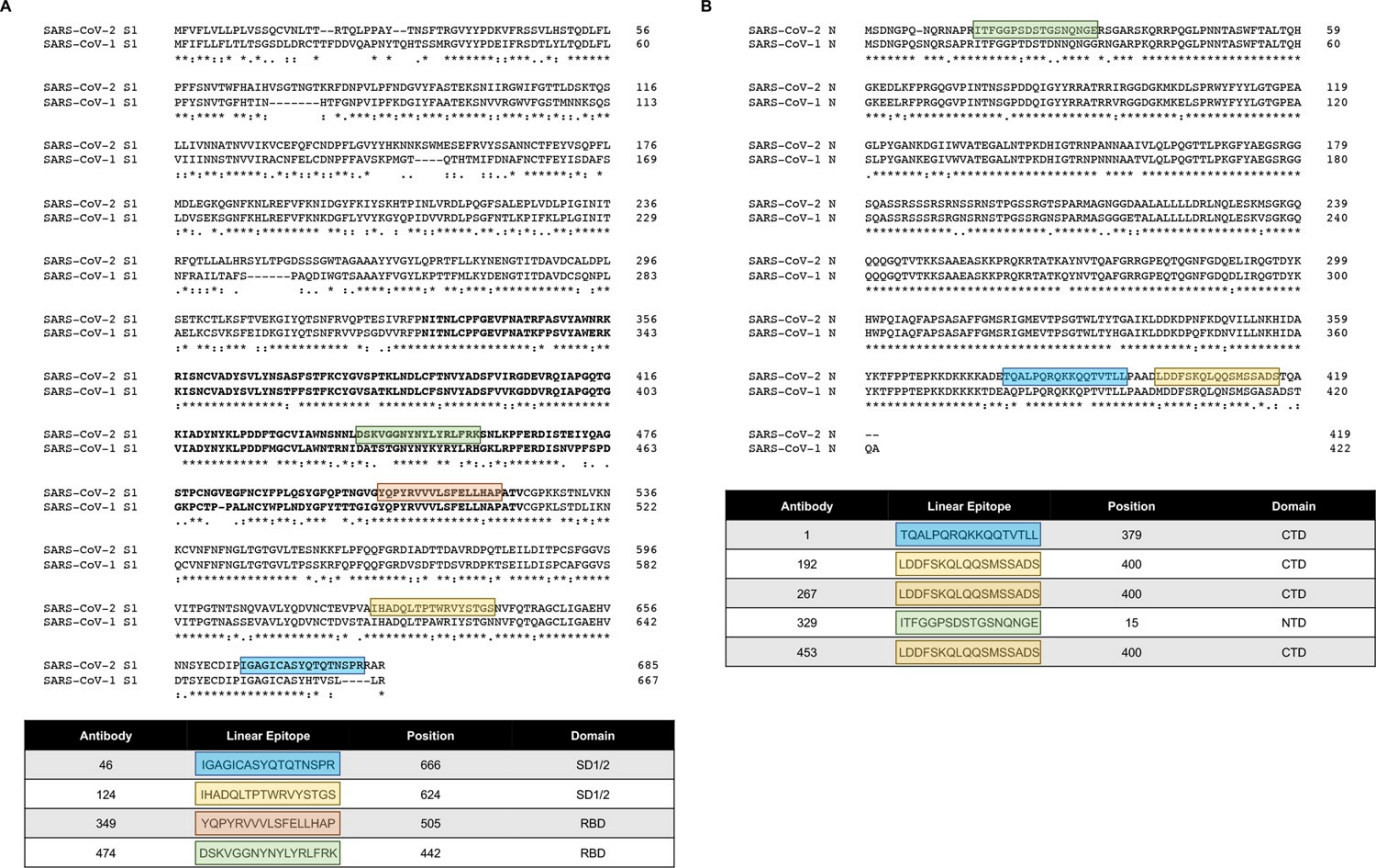
SARS-CoV-2 S1 and N protein alignment and linear epitope mapping of the mAbs. (A) Clustal Omega-generated amino acid sequence alignment of SARS-CoV-2 S1 and SARS-CoV-1 S1. The epitope recognized by each mAb is indicated by the highlighted boxes, the position of the epitope is noted. SD1/2, subdomain 1/2. RBD, receptor binding domain. The amino acids representing the RBD are in bold. (B) Clustal Omega-generated amino acid sequence alignment of SARS-CoV-2 N and SARS-CoV-1 N. The epitope recognized by each mAb is indicated by the highlighted boxes, the position of the epitope is noted. CTD, C terminal domain. NTD, N terminal domain. *, single, fully conserved residue;:, conservation between groups of strongly similar properties (i.g., those scoring >0.5 in the Gonnet PAM 250 matrix); •, conservation between groups of weakly similar properties (i.e., those scoring ≤0.5 in the Gonnet PAM 250 matrix).

To define antibody competition groups, we performed linear epitope mapping via peptide scanning ELISAs with candidate mAbs. Libraries of SARS-CoV-2 S1 and N overlapping peptides were coated in 96-well plates and incubated with each mAb. After washing, positive signals were detected using an anti-mouse IgG antibody coupled to horseradish peroxidase for signal development. Linear epitope mapping revealed that the SARS-CoV-2 S1 mAbs recognize different epitopes within the protein (Fig 3A). mAbs 46 and 124 recognized epitopes within subdomains 1/2 (SD1/2) of S1, while mAbs 349 and 474 recognized an epitope within the receptor binding domain (RBD) of S1. We confirmed recognition of these SARS-CoV-2 S1 domains by immunoblot analysis (S3 Fig). The SARS-CoV-2 N mAbs either recognized epitopes within the C-terminal domain (CTD) or N-terminal domain (NTD) of N (Fig 3B). mAbs 192, 267, and 453 were clones and recognized the same epitope within the CTD, whereas mAb 1 recognized a different epitope within the CTD, and mAb 329 recognized an epitope within the NTD.

**Fig 4:**
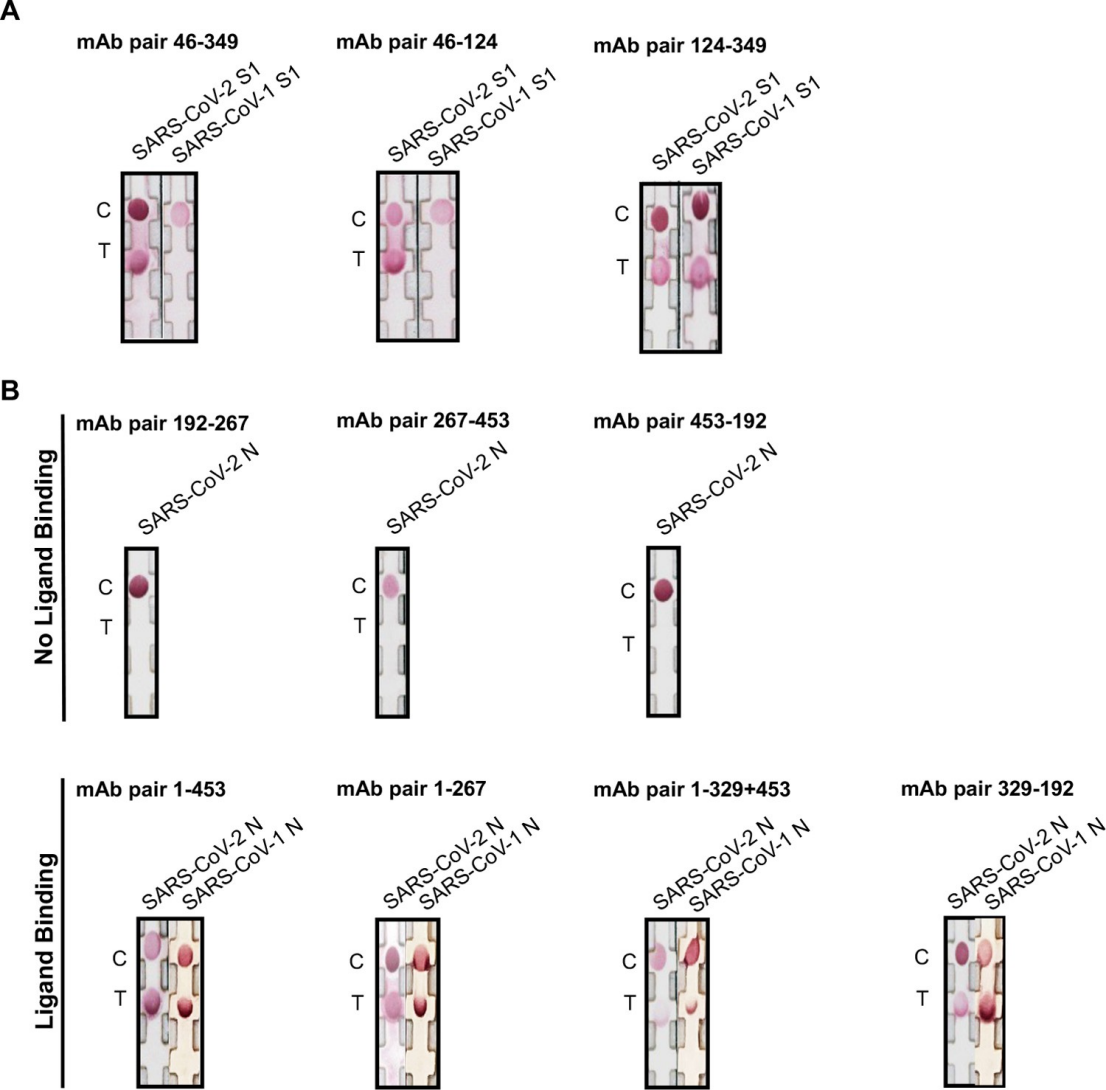
SARS-CoV-2 S1 and N interactions in lateral flow immunochromatography. mAbs were either conjugated to gold nanoparticles or adsorbed onto the nitrocellulose membrane. Different combinations were tested to distinguish between specific and cross-reactive detection and indicate differential epitope binding. SARS-CoV-1/2 S1 or SARS-CoV-1/2 N, at final concentrations of 125 ng/ml, were allowed to react with the rapid antigen test. (A) mAb pairs 46–349 and 46–124 specifically recognize SARS-CoV-2 S1, whereas mAb pair 124–349 recognize both SARS-CoV-2 S1 and SARS-CoV-1 S1. (B) Ligand binding is not observed in rapid tests composed of mAb pairs 192–267, 267–453, and 453–192. However, ligand binding occurs with mAb combinations 1–453, 1–267, 1–329+453, and 329–192; these combinations detect both SARS-CoV-1 and SARS-CoV-2 N. C, control. T, test.

The epitope mapping experiments helped to explain the observed SARS-CoV-2 S1 specificity versus SARS-CoV-2 and SARS-CoV-1 S1 and N cross-reactivity, and the ability to detect SARS-CoV-2 N by various mAb pairs, while other pairs were unable to detect the protein. It appeared that mAb 46 conferred specificity towards SARS-CoV-2 S1 given it recognized an epitope that contains 3 amino acids that was not present in SARS-CoV-1 S1. When mAb 46 was paired with mAbs 349 or 124, no detection of SARS-CoV-1 S1 was observed (Fig 4A). However, when paired, mAbs 124 and 349 recognized both SARS-CoV-1 and SARS-CoV-2 S1 proteins (Fig 4A). Furthermore, the SARS-CoV-2 N mAbs 192, 267, and 453 recognized the same epitope. As such, these mAbs, when paired, were unable to detect the N protein in lateral flow chromatography (Fig 4B). In contrast, mAbs 192, 267, and 453 were able to detect SARS-CoV-2 N when paired with mAbs that did not recognize the same epitope (Fig 4B). Additionally, given the similarity in epitopes recognized by the N mAbs, the rapid antigen tests for SARS-CoV-2 N, also cross-reacted with SARS-CoV-1 N (Fig 4B). Altogether, the epitope mapping data provided an important framework for understanding the mechanisms of SARS-CoV-2 S1 and N detection in gold conjugate lateral flow immunochromatography.

### Performance of the SARS-CoV-2 N rapid antigen test using patient nasal samples

**Fig 5:**
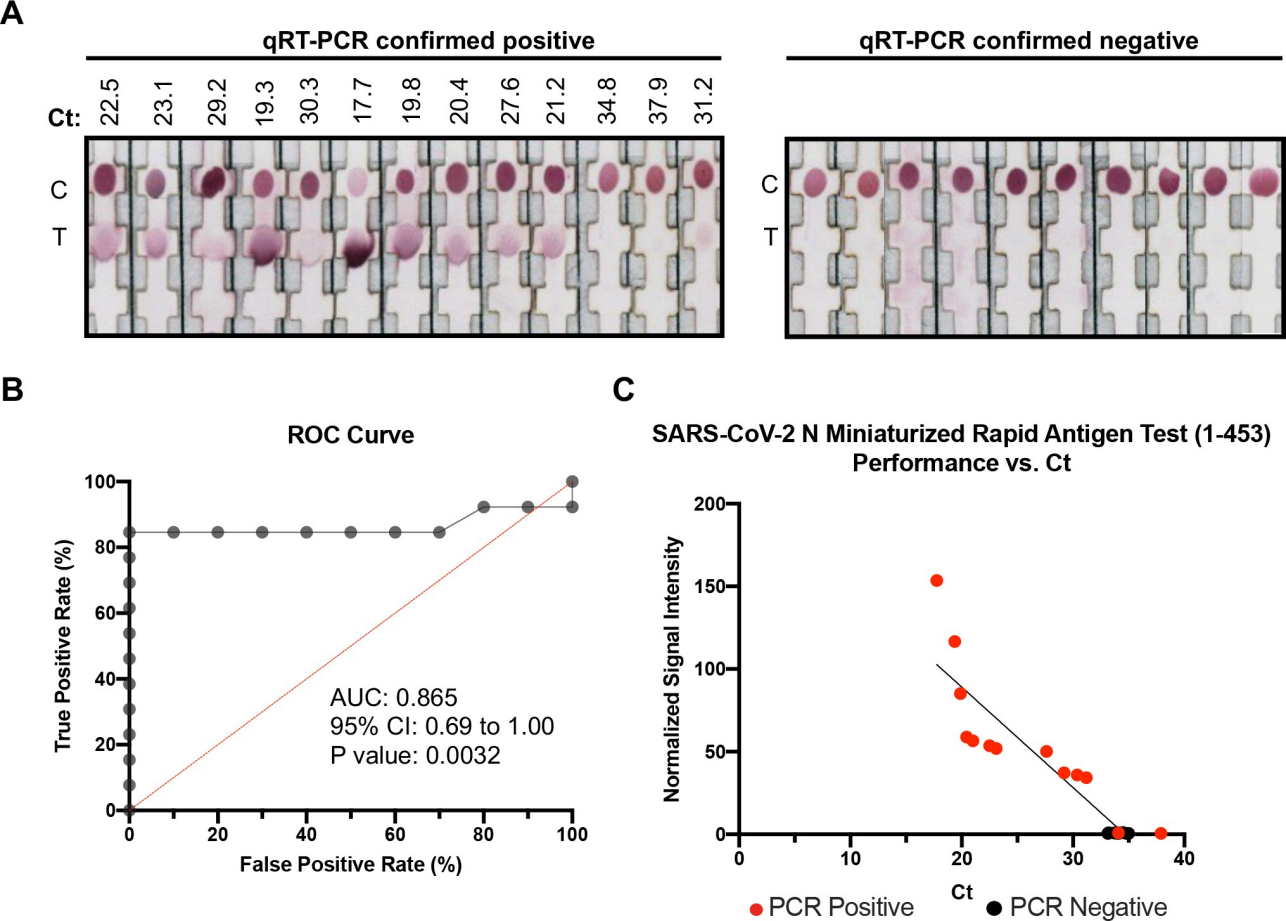
SARS-CoV-2 N rapid antigen test performance. (A) Nasal swab specimen (50 μl) collected from qRT-PCR confirmed positive patients (n = 13) and negative patients (n = 10) were allowed to react with a SARS-CoV-2 N rapid antigen test composed of mAbs 1–453. Ct, Cycle threshold value. C, control. T, test. (B) Receiver operator characteristic (ROC) curve of SARS-CoV-2 N rapid antigen test. Test performance is demonstrated in terms of true positive rate (sensitivity) versus false positive rate (1-specificity). AUC, area under the curve. CI, confidence interval. (C) Normalized signal intensity was compared to the qRT-PCR-derived sample cycle threshold (Ct) value. Points are plotted with a linear regression line of y = -6.378x + 21 and p < 0.0001 and R^2^ = 0.847.

We retrospectively tested the performance of a candidate SARS-CoV-2 N rapid antigen test using 23 nasal swab specimens, collected during the acute phase of COVID-19. Thirteen samples were confirmed RT-PCR positive for SARS-CoV-2 and 10 were RT-PCR negative. 50μl of each nasal swab specimen was applied to the SARS-CoV-2 N rapid antigen test and allowed to react for 15 minutes, after which normalized signal pixel intensities were image captured and scored. The SARS-CoV-2 N rapid antigen test detected 11 out of 13 positive specimens (84.6% sensitivity) with Ct values ranging between 17.7 and 31.2; the test was unable to detect samples with Ct values between 34.8 and 37.9. The test did not detect a signal with any of the negative specimens (100% specificity) (Fig 5A).

A Receiver Operator Characteristic (ROC) curve illustrates the performance of the SARS-CoV-2 N rapid antigen test as a function of the discrimination threshold, plotted as sensitivity versus 1—specificity. The area under the curve (AUC) is a proxy of test performance, where 1 represents a perfect test, and 0.5 represents a random predictor. We measured an AUC of 0.865 for the SARS-CoV-2 N rapid antigen test (Fig 5B). To understand how the signal pixel intensity results change with patient viremia, the data was plotted against RT-PCR Ct values. Ct values represent the number of RT-PCR cycles at which the generated fluorescence crosses a threshold; the lower the Ct value, the fewer replication cycles are needed to detect the virus. The signal pixel intensity results were inversely related to the Ct value, with a linear regression line of y = -6.378x + 21 and p < 0.0001 and R^2^ = 0.847 (Fig 5C). Our results demonstrate that the SARS-CoV-2 N rapid antigen test is optimized to detect SARS-CoV-2 during the early phase of COVID-19 where Ct values are generally observed below 30.

## Discussion

Diagnosis of COVID-19 by RT-PCR is the clinical gold standard for laboratory-based diagnosis, yet remains impractical especially in resource-limited settings due to its high cost and slow turnaround times. Antigen tests for the rapid detection of SARS-CoV-2 provide important benefits for public health systems managing outbreaks; these tests typically have a retail price between $3–10 and can be self-administered, thereby allowing wide-scale community surveillance and immediate quarantining of infected individuals. Antigen tests have further applications for patient outcomes, where immediate results can help inform clinical decisions. While early and accurate detection of SARS-CoV-2 is essential, the process can be complicated by co-circulating pathogens (e.g., SARS-CoV-1, influenza A/B, rhinovirus, respiratory syncytial virus) that may clinically present with similar symptoms. Therefore, sensitive and specific diagnostics for accurate identification of COVID-19 are urgently needed. To date, 24 of 25 antigen-based tests for SARS-CoV-2 detection that have obtained an Emergency Use Authorization from the FDA cross-react with SARS-CoV-1, and only 8 tests can be self-administered in the home (S1 Table).

This study describes prototype rapid antigen tests that specifically detect the S1 and N proteins of SARS-CoV-2 with or without cross-reaction to SARS-Cov-1. Throughout the pandemic, numerous SARS-CoV-2 variants have emerged with varying infection and disease dynamics. A rapid test that can specifically identify SARS-CoV-2 and its variants, but not other coronaviruses that have emerged in the past (i.e, SARS-CoV-1, MERS, etc.) is an important public health tool for outbreak response. Despite the high percentages of homology and identity among the SARS-CoV-1/2 N proteins, our mAb screening strategy identified pairs that detected and/or distinguished SARS-CoV-2. First, we immunized mice with recombinant S1 and N proteins expressed by eukaryotic cells. Second, we analyzed relative binding to various S1 and N proteins by ELISA using the hybridoma supernatants, followed by flow cytometric analysis to confirm specific and cross binding to native proteins in virus-infected cells (Figs 1, S2, Table 1). Interestingly, SARS-CoV-2 S1 spleen-derived mAbs (B cell isolation and cell fusion at 105 days post-immunization) were less unique to SAR-CoV-2 S1 compared to lymph node-derived mAbs (B cell isolation and cell fusion at 23 days after immunization). Additionally, N mAbs pairs had better LoDs. Third, we performed systematic pairwise immunochromatographic screening of candidate mAbs to identify pairs that recognize SARS-CoV-2 (S3 Fig). Our mAb screening approach has previously been utilized to identify specific mAbs for dengue viruses 1–4, Zika virus, and chikungunya virus [25,26].

Eleven complementary combinations of mAbs (S1 pairs: 46–474, 124–474, 46–349, 124–349, 303–349, 124–46, and 474–349; N pairs: 1–453, 1–267, 1+329–453, and 329–192) were used to develop rapid antigen test prototypes. Laboratory testing using recombinant proteins demonstrated that the SARS-CoV-2 S1 and N rapid antigen tests had LoDs that ranged between 4.89–9.06 ng/ml and 0.76–6.95 ng/ml, respectively (Fig 2A and 2B, Table 2). The SARS-CoV-2 S1 rapid antigen tests resulted in much higher LoDs and as such were not considered sufficiently sensitive. However, the LoDs of two SARS-CoV-2 N antigen tests were well within the diagnostic range. A rapid antigen test composed of mAbs 1 and 453 was subsequently validated using contrived nasal swab specimen dilutions (Fig 2C). We evaluated the performance in a low number of RT-PCR positive and negative nasal swab specimens; 50 μl of each specimen was processed by the rapid antigen test. The SARS-CoV-2 N rapid antigen test performed with 84.6% sensitivity (11/13) and 100% specificity (10/10) (Fig 5A). The rapid antigen test was able to detect all RT-PCR confirmed positive specimens that contained Ct values below 31.2, suggesting that the test is optimized to detect COVID-19 during the acute, infectious phase [19,21,22,24,27–31].

Epitope mapping experiments in conjunction with immunochromatography experiments helped elucidate competitive groups of mAbs (Figs 3A and 4A). Moreover, all SARS-CoV-2 N rapid tests all cross-reacted with SARS-CoV-1 (Fig 4B) due to greater than 70% amino acid homology between SARS CoV-1 and SARS-CoV-2. This is consistent with our mAb screening data given that N mAbs recognize both SARS-CoV-1/2 N proteins from both SARS viruses (Fig 1). Interestingly, N mAbs that recognize the same epitope do not result in ligand binding (Fig 3B and 4B). A likely explanation is that the mAbs compete with each other for the same epitope, therefore, complex formation between the gold conjugate, test line mAbs, and the target protein does not occur. However, using N mAbs that recognize different epitopes does result in complex formation. These results provide a framework for understanding the mechanisms of specific protein interactions in lateral flow chromatography.

Point-of-care and self-administered rapid tests have been proposed as important public health tools to help combat the pandemic [32–34]. Our study provides a foundation to develop antigen-based tests that can be implemented for diagnosis and surveillance of COVID-19. Our rapid test development utilized a high-throughput screening protocol based on ELISAs, FACS analysis, and lateral flow immunochromatography. These steps, along with quantitative image analysis for candidate antibody pairs can enable efficient laboratory validations of antigen tests. The low-cost and simplicity of the design of our rapid tests can meet the high demands for diagnosis and surveillance in hospitals, schools, workplaces, and airports around the world. Because the rapid antigen tests can be self-administered and results interpreted within 15 minutes, this format is ideal for serial testing. Studies have shown that higher frequency testing should be prioritized over accuracy, making the ease-of-use and accessibility of these antigen-based tests critical for disease control [19,21,22,24,35]. While our sample size is limited, our test currently performs with similar accuracy to the currently available at-home tests, rendering future studies vital for further validating the rapid test for widespread use. In future studies, evaluation of the rapid test should include more samples collected from individuals during the asymptomatic and symptomatic phases of COVID-19. While most rapid antigen tests currently on the market are approved for use with nasal swab specimens, other sample types including oropharyngeal swab specimens or saliva will be important to evaluate. A number of antigen tests have been approved for self-testing. Determining the efficacy of self-testing using the rapid test presented in this study will help elucidate whether the test in its particular format can be used outside of a profession setting. In particular, evaluation of self-testing using the rapid antigen test should include a variety of geographies, including low and middle income regions, or settings with higher COVID-19 incidence.

Resource-limited countries throughout the tropics and subtropics have been found to be among the most frequently and most severely affected by the COVID-19 pandemic [36]. Because of important COVID-19-specific response efforts, other health services have been negatively impacted, especially in these regions. Disruptions of community-based interventions, delays in diagnosis, treatment and care, discontinuation of routine surveillance for other neglected tropical diseases (NTDs), and diversion of financial resources elsewhere are among the health services that are impacted. As a consequence of these disruptions, projected increased burdens of NTDs, in terms of mortality and morbidity are expected, as well as delays in elimination and eradication of NTDs more broadly. The antibody screening method proposed in this study can serve as a template for the development and identification of mAbs that target other NTDs. These mAbs can then be translated into low-cost rapid tests that can help alleviate some of the burden brought on by COVID-19.

It is important that all populations have access to a repertoire of high performing, low-cost diagnostics. Rapid antigen-based tests are valuable because they easily detect the viral proteins generated during acute disease. More broadly, rapid tests, PCR assays, and serology tests are all necessary to meet various care and surveillance needs in different contexts. Taken together, this study describes the development and validation of specific and sensitive SARS-CoV-2 rapid antigen tests, which we believe is an important point-of-care and screening tool to enable more efficient and equitable disease prevention and societal re-opening.

## Supporting information

S1 Fig Relative binding of SARS-CoV-2 S1 and N mAbs. mAb clones were harvested from the hybridomas of SARS-CoV-2 spike subunit 1 (S1) or nucleocapsid (N) immunized mice. The monoclonal antibodies (mAbs) were screened through ELISA to analyze the relative binding to recombinant S1 and N proteins. Binding is quantified as the OD_450_ and normalized as Fold Above Background. (A) ELISA using hybridoma supernatants from the lymph nodes of mouse EB017 infected with SARS-CoV-2 S1. Hybridoma supernatants were tested with S1 from SARS-CoV-2, SARS-CoV-1, MERS, NL63, 229E, HKU1, and OC43 to evaluate cross-reactivity. (B) ELISA using hybridoma supernatants from the spleen of mouse EB024 infected with SARS-CoV-2 S1. Hybridoma supernatants were tested with S1 from SARS-CoV-2, SARS-CoV-1, MERS, NL63, 229E, HKU1, and OC43 to evaluate cross-reactivity. (C) ELISA using hybridoma supernatants from the lymph nodes of mouse EB025 infected with SARS-CoV-2 N. Hybridoma supernatants were tested with S1 from SARS-CoV-2 and SARS-CoV-1 to evaluate cross-reactivity.
(TIF)

S2 Fig Flow cytometric analysis of SARS-CoV-2 mAbs. Hybridoma supernatants were used to stain permeabilized Vero cells infected with SARS-CoV-2, the human coronaviruses 229E or OC43, or non-infected cells as a control (Mock). FACS analysis was performed using (A) SARS-CoV-2 S1 mAbs derived from mouse EB017 lymph nodes, (B) SARS-CoV-2 S1 mAbs derived from mouse EB024 lymph nodes, (C) SARS-CoV-2 N mAbs derived from mouse EB025 lymph nodes. Fluorescence is normalized as Fold Above Background.
(TIF)

S3 Fig Combinatorial Dipstick Analysis. (A) SARS-CoV-2 S1 mAbs and (B) SARS-CoV-2 N mAbs were tested in a matrix for interaction with SARS-CoV-2 S1 or N and without added antigen as a control (—). mAbs were either conjugated to the nanoparticle or placed onto the nitrocellulose paper. SARS-CoV-2 S1 or SARS-CoV-2 N, at final concentrations of 125 ng/ml, were allowed to react with the rapid antigen tests. The pairwise immunochromatography signal intensities are scored as low binding (1, >201 normalized grey scale pixel intensity), medium binding (2, 141–200 normalized grey scale pixel intensity), or high binding (3, <140 normalized grey scale pixel intensity), with a majority of the combinations presenting with no signal (white). Grey boxes represent combinations not tested.
(TIF)

S4 Fig Western blot analysis using SARS-CoV-2 S1 mAbs. Immunoblots were performed using SARS-CoV-2 spike protein immunostained with (A) mAb 46, (B) mAb 124, (C) mAb 349, and (D) mAb 474 to elucidate mAb binding to spike subunit 1 (S1), spike subunit 2 (S2), or the receptor binding domain (RBD). kDa, kilodalton.
(TIF)

S1 Table SARS-CoV-2 rapid antigen tests with emergency use authorizations (EUA). (DOCX)
