# Supplementary figures and images for: Monoclonal antibody pairs against SARS-CoV-2 for rapid antigen test development
Source: PLoS Negl Trop Dis. 2022 Mar 31;16(3):e0010311. doi: 10.1371/journal.pntd.0010311 (PMC9004783; doi:10.1371/journal.pntd.0010311)

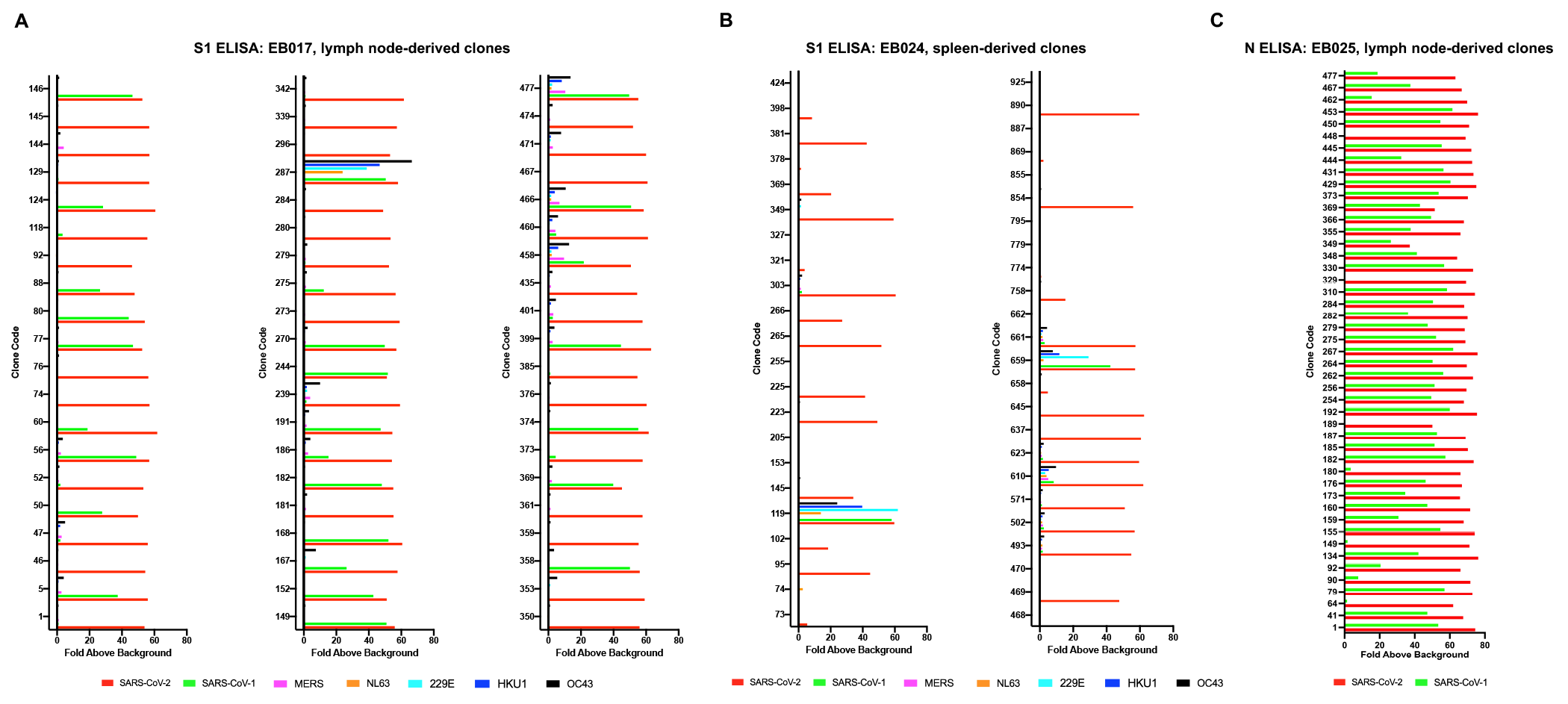

Supplement: S1 Fig — The monoclonal antibodies (mAbs) were screened through ELISA to analyze the relative binding to recombinant S1 and N proteins. Binding is quantified as the OD450 and normalized as Fold Above Background. (A) ELISA using hybridoma supernatants from the lymph nodes of mouse EB017 infected with SARS-CoV-2 S1. Hybridoma supernatants were tested with S1 from SARS-CoV-2, SARS-CoV-1, MERS, NL63, 229E, HKU1, and OC43 to evaluate cross-reactivity. (B) ELISA using hybridoma supernatants from the spleen of mouse EB024 infected with SARS-CoV-2 S1. Hybridoma supernatants were tested with S1 from SARS-CoV-2, SARS-CoV-1, MERS, NL63, 229E, HKU1, and OC43 to evaluate cross-reactivity. (C) ELISA using hybridoma supernatants from the lymph nodes of mouse EB025 infected with SARS-CoV-2 N. Hybridoma supernatants were tested with S1 from SARS-CoV-2 and SARS-CoV-1 to evaluate cross-reactivity. (TIF) [file pntd.0010311.s001.tif]

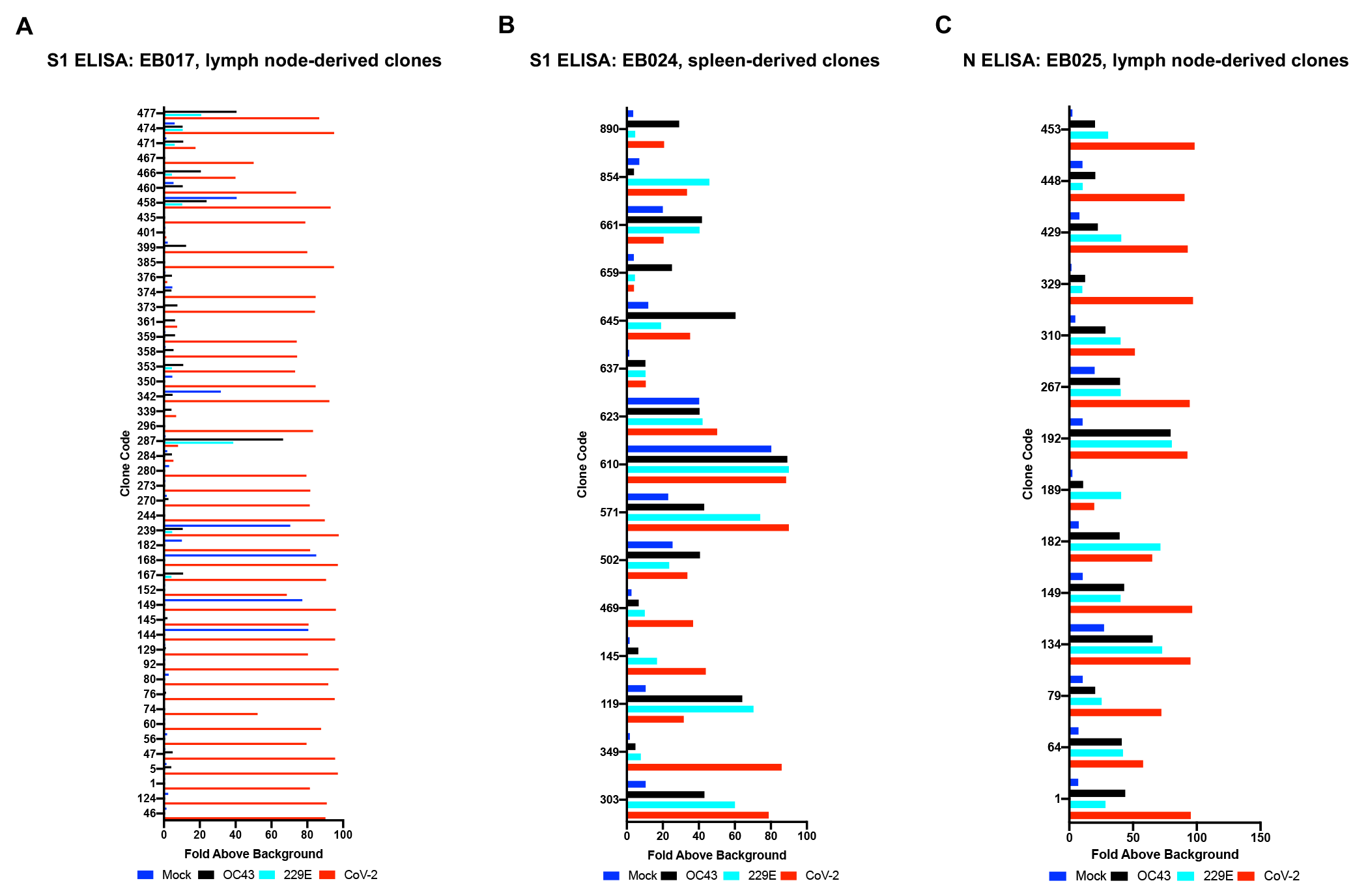

Supplement: S2 Fig — Hybridoma supernatants were used to stain permeabilized Vero cells infected with SARS-CoV-2, the human coronaviruses 229E or OC43, or non-infected cells as a control (Mock). FACS analysis was performed using (A) SARS-CoV-2 S1 mAbs derived from mouse EB017 lymph nodes, (B) SARS-CoV-2 S1 mAbs derived from mouse EB024 lymph nodes, (C) SARS-CoV-2 N mAbs derived from mouse EB025 lymph nodes. Fluorescence is normalized as Fold Above Background. (TIF) [file pntd.0010311.s002.tif]

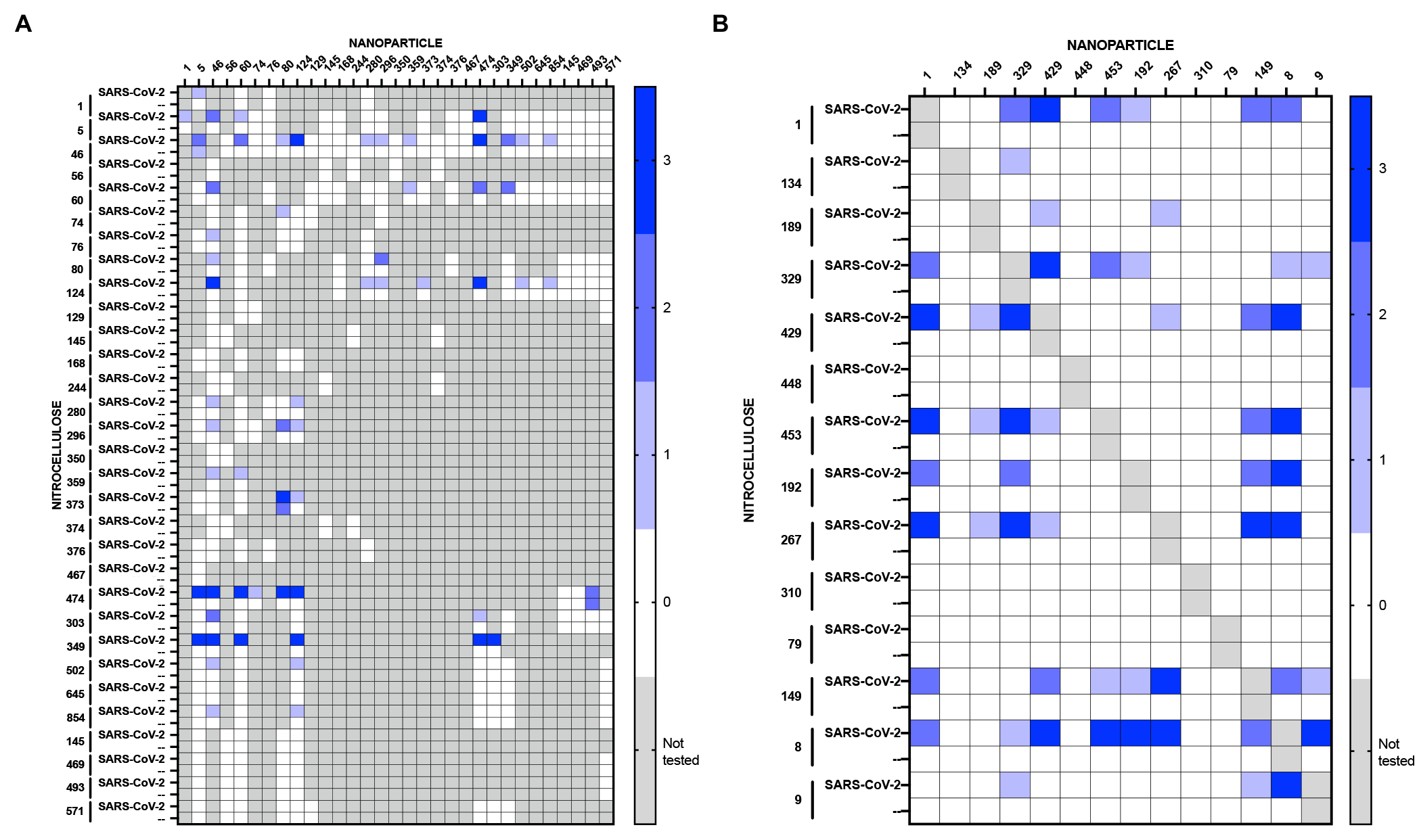

Supplement: S3 Fig — (A) SARS-CoV-2 S1 mAbs and (B) SARS-CoV-2 N mAbs were tested in a matrix for interaction with SARS-CoV-2 S1 or N and without added antigen as a control (—). mAbs were either conjugated to the nanoparticle or placed onto the nitrocellulose paper. SARS-CoV-2 S1 or SARS-CoV-2 N, at final concentrations of 125 ng/ml, were allowed to react with the rapid antigen tests. The pairwise immunochromatography signal intensities are scored as low binding (1, >201 normalized grey scale pixel intensity), medium binding (2, 141–200 normalized grey scale pixel intensity), or high binding (3, <140 normalized grey scale pixel intensity), with a majority of the combinations presenting with no signal (white). Grey boxes represent combinations not tested. (TIF) [file pntd.0010311.s003.tif]

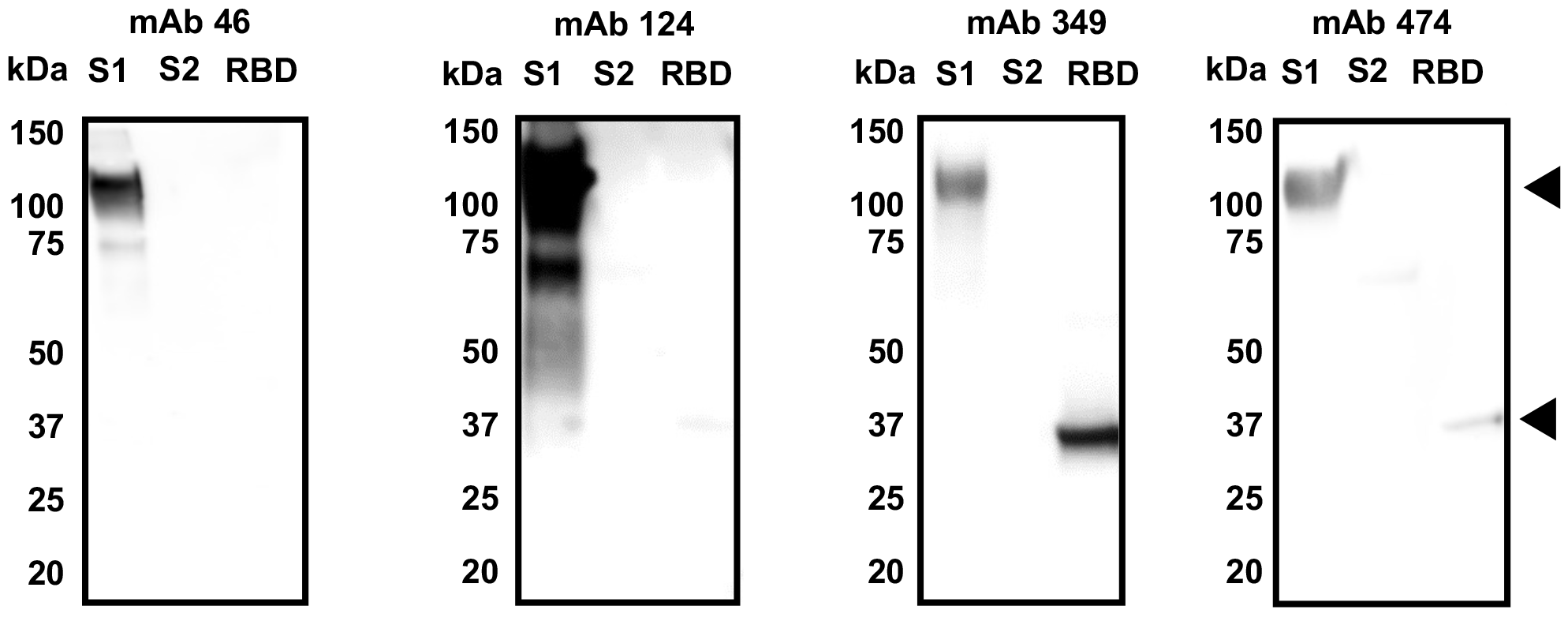

Supplement: S4 Fig — Immunoblots were performed using SARS-CoV-2 spike protein immunostained with (A) mAb 46, (B) mAb 124, (C) mAb 349, and (D) mAb 474 to elucidate mAb binding to spike subunit 1 (S1), spike subunit 2 (S2), or the receptor binding domain (RBD). kDa, kilodalton. (TIF) [file pntd.0010311.s004.tif]
