# Supplementary material for: Monoclonal antibody pairs against SARS-CoV-2 for rapid antigen test development
Source: PLoS Negl Trop Dis. 2022 Mar 31;16(3):e0010311. doi: 10.1371/journal.pntd.0010311 (PMC9004783; doi:10.1371/journal.pntd.0010311)
Supplement: S1 Table — (DOCX) [file pntd.0010311.s005.docx]

| **Entity** | **Diagnostic** | **Date of EUA Issue** | **Authorized Setting(s)^1^** | **Cross-Reactivity with SARS-CoV-1** |
| --- | --- | --- | --- | --- |
| **Salofa Oy** | Sienna-Clarity COVID-19 Antigen Rapid Cassette | 05/20/2021 | H, M, W | Cross-Reactive |
| **PHASE Scientific** | INDICAID COVID-19 Rapid Antigen Tests | 07/28/2021 | H, M, W |  |
| **QIAGEN GmbH** | QIAreach SARS-CoV-2 Antigen | 08/05/2021 | H, M |  |
| **GenBody Inc.** | GenBody COVID-19 Ag | 09/24/2021 | H, M, W |  |
| **ANP Technologies, Inc.** | NIDS COVID-19 Antigen Rapid Test Kit | 09/24/2021 | H, M, W |  |
| **Xtrava Health** | SPERA COVID-19 Ag Test | 09/24/2021 | H, M, W |  |
| **ACON Laboratories, Inc.** | Flowflex COVID-19 Antigen Home Test | 10/19/2021 | Home, H, M, W |  |
| **Celltrion USA, Inc.** | Sampinute COVID-19 Antigen MIA | 10/23/2020 | H,M |  |
|  | Celltrion DiaTrust COVID-19 Ag Rapid Test | 10/21/2021 | Home, H,M,W |  |
| **Abbott Diagnostics** | BinaxNOW COVID-19 Antigen Self-Test | 03/31/2021 | Home, H,M,W |  |
| **Access Bio, Inc.** | CareStart COVID-19 Antigen test | 10/08/20 | H,M,W |  |
| **DiaSorin, Inc.** | LIAISON SARS-CoV-2 Ag | 03/26/21 | H,M |  |
| **Quidel Corporation** | QuickVue At-Home OTC COVID-19 Test | 03/31/2021 | Home, H, M, W |  |
|  | Sofia 2 Flu + SARS Antigen FIA | 10/02/2020 | H,M,W |  |
| **Ellume Limited** | Ellume COVID-19 Home Test | 12/15/2020 | Home, H,M,W |  |
| **InBios International, Inc.** | SCoV-2 Ag Detect Rapid Test | 05/06/2021 | H,M,W |  |
| **Luminostics, Inc.** | Clip COVID Rapid Antigen Test | 12/07/2020 | H,M,W |  |
| **LumiraDx UK Ltd.** | LumiraDx SARS-CoV-2 Ag Test | 08/18/2020 | H,M,W |  |
| **Ortho Clinical Diagnostics, Inc.** | VITROS Immunodiagnostic Products SARS-CoV-2 Antigen Reagent Pack | 01/11/2021 | H,M |  |
| **Princeton BioMeditech Corp.** | Status COVID-19/Flu | 02/24/21 | H,M,W |  |
| **Qorvo Biotechnologies, LLC.** | Omnia SARS-CoV-2 Antigen Test | 04/13/2021 | H,M |  |
| **Quanterix Corporation** | Simoa SARS-CoV-2 N Protein Antigen Test | 01/05/2021 | H,M |  |
| **Orasure Technologies, Inc.** | IteliSwab COVID-19 Rapid Test | 06/04/21 | Home, H, M, W |  |
| **Becton, Dickinson and Company (BD)** | BD Veritor System for Rapid Detection of SARS-CoV-2 | 08/24/2020 | Home, H,M,W |  |
|  | BD Veritor System for Rapid Detection of SARS-CoV-2 & Flu A+B | 03/24/2021 | Home, H,M,W | Non-Cross-Reactive |
